# Highly reusable and superhydrophobic spongy graphene aerogels for efficient oil/water separation

**DOI:** 10.1038/s41598-017-07583-0

**Published:** 2017-08-02

**Authors:** Yuanzheng Luo, Shenlin Jiang, Qi Xiao, Chuanliang Chen, Buyin Li

**Affiliations:** Key Laboratory of Electronic information functional material of Ministry of Education, School of Optical and Electronic Information, Huazhong University of Science and Technology, Wuhan, Hubei 430074 China

## Abstract

Graphene aerogels (GAs) are three-dimensional (3D) graphene sponges with unique wettability and have demonstrated the potential for reducing contamination from oil spills and chemical accidents. Herein, we report new polyurethane (PU) sponge-reinforced GAs with low surface energy, high sorption capacity and excellent recyclability for use as efficient oil sorbents. Spongy graphene aerogels (SGAs) with a hierarchical porous morphology were produced by simply freeze-casting reduced graphene oxide (rGO) to form compacted macroscale sponges. This novel micro-structure benefits from the advantages of embedded graphene and presents reversible large-strain deformation (90%), high compressive strength (63 kpa) and viscoelastic stability. These superior properties, in addition to super-hydrophobicity, endow the aerogels with excellent recyclability without deteriorating the oil absorption performance. Furthermore, SGA has selective and high-volume absorbability (>100%) and can efficiently separate oil from water under continuous pumping action. The excellent absorption performance and robust mechanical properties make this graphene material promising for the large-scale recovery of spilled oil.

## Introduction

Fossil fuels such as crude oil are the bedrock of modern civilization and are still in very high demand. However, the exploitation and utilization of such fuels have led to oil spill accidents contaminating the ecosystem^1^. As fossil fuel infrastructures expand, more spills and leaks of oil pollutants occur. Therefore, the separation of oil from water bodies has attracted particular attention over the years. Among the environmental remediation strategies for oil spills, mechanical remediation using sorbent materials is considered one of the most efficient and cost-effective methods^2,3^. Up to now, a variety of sorbent materials with different porosities and surface chemistries, such as natural organic materials, inorganic mineral products, synthetic membranes, microporous polymeric materials and carbon-based nanomaterials, have been used for oil recovery. Due to their high accessible pore volume and unique wettability as well as their modification possibilities, microporous polymers and advanced carbon nano-materials have obvious advantages over other absorbents. For the latter, various carbon-based aerogels with extraordinary porosities and hydrophobicity play an important role in the treatment of petroleum pollution^4–7^. In particular, research into free-standing graphene aerogels possessing specific functionalities and architectures produced by a reduction self-assembly process are rapidly evolving with new drying techniques and applications^5,8–11^. The assembly of graphene into three-dimensional (3D) porous monoliths retains the intrinsic advantages of both components which enhances the potential of the material in practical oil-absorption applications^12^. Liu *et al*.^7^ developed a simple directional freezing approach to produce an anisotropic oil absorbent that retains its initial height without attenuation after 20 compression-recovery cycles. Zhang *et al*.^13^ presented an oil-water interface assembly strategy for fabricating cellular GAs, the as-prepared GAs filled with n-hexane can be easily vacuated over dozens of cyclic compressions while maintaining an absorbability over 90%. Although most superelastic GAs can be readily fabricated by cost-efficient ice-templating methods, the thickness of GA is inevitably reduced over more compression cycles^7,8^. The durability of GAs is considered a crucial characteristic for the efficient mechanical extraction of spilled oil.

On the other hand, elastomeric graphene composites, which introduce graphene properties such as elasticity and strength into microporous polymers have the potential to improve the reusability of neat graphene absorbents. Therefore, numerous synthetic routes for polymer-reinforced graphene composites including solution dip-coating^14,15^, chemical vapor deposition (CVD)^16,17^, cross-linked assembly^18^, and emulsion-based assembly^19^ have been reported to fabricate hydrophobic and reusable absorbents. Among them, dip-coating is a facile one-step process, and microporous polymers coated with the graphene “skins” have recently attracted intense research interest for oil-water separation recently. These graphene-coated materials which have unique wettability have been synthesized using commercially available foams such as melamine^20,21^, polydimethylsiloxane (PDMS)^22^ and polyurethane sponges (PUS)^23,24^. Yao *et al*.^15^ reported that the fractured microstructure of graphene-coated PUS (GCS) resulting from pre-compression was critical in enhancing the elastic properties and cyclicity. Dai *et al*.^25^ present a graphene melamine foam with enhanced flexibility and hydrophobicity, which had a water contact-angle (WCA) of 127° due to the increased surface roughness from the same pre-loading treatment. As a result, the pre-compaction of the polymer matrices is an ideal and simple route for mass production of advanced absorbents owing to its easy handling. In addition, thermoset microporous polymers, such as PUS, are notoriously difficult to recycle. Large quantities of waste sponges are generated daily in plastic production plants^26^. Therefore, exploring applications of recyclable graphene/PUS materials is a greener way to solve the problem of oil pollution.

Herein, we use sponge waste to prepare spongy graphene aerogels (SGAs) via pre-compaction and ice-templated assembly. The resulting absorptive graphene/PUS material has a hierarchical 3D structure and excellent properties, arising from both components, owing to the cellular graphene aerogels assembled onto the PUS skeleton. To fully understand the significant advantages of our SGA over conventional graphene-coated sponges (GCS). The dynamic mechanical thermal analysis (DMTA) was performed to elucidate the effect of the buckled and hierarchical porous structure. Furthermore, the sorption capacity and recyclability of SGA were examined, and encouraging results obtained. For the removal of petroleum products, the nested porous structure of SGA contributed to a much higher volume-based absorption capacity than that in other reported foams. Additionally, the highly selective properties of SGA make the separation of oil-water mixtures more efficient, especially under a continuous pumping action. The high porosity and super-hydrophobic surface (WCA of 152°)of SGA are believed to play critical roles in the high sorption capacity and excellent recyclability. Finally, we also investigated effect of different concentrations of the graphene oxide (GO) hydrogel precursor on surface wettability and hydrophobic mechanism.

## Results

### Morphology of the SGA

**Figure 1:**
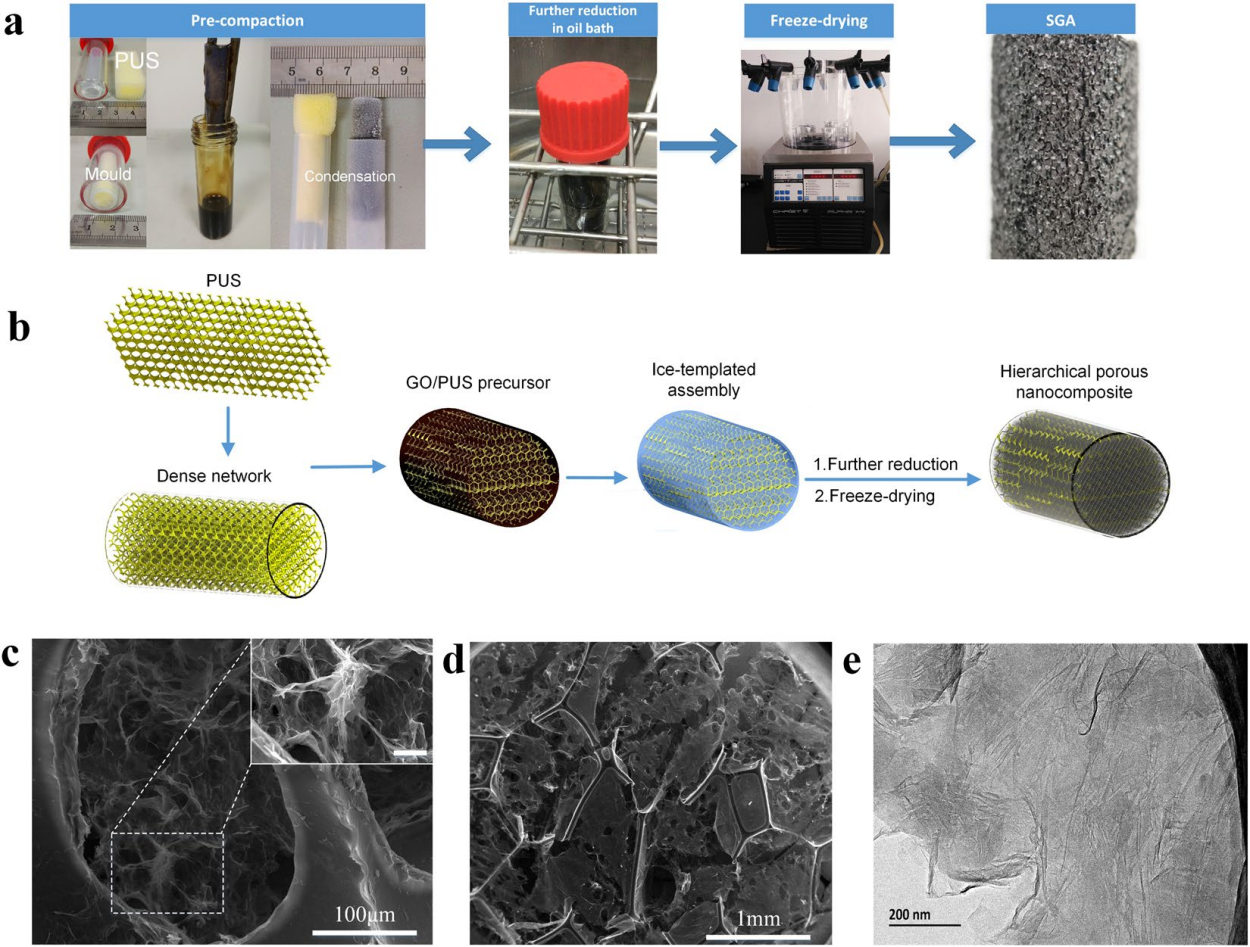
(**a**) Digital images of the SGA fabrication process. (**b**) A schematic representation of the different stages during the formation of the hierarchical porous structure; (**c**) SEM images of the nested porous structure (inset scale bar is 10 *μ*m). (**d**) Magnified image of hierarchical structure assembled from rGO/sponge from the Ice-templating method. (**e**) The transmission electron microscopy (TEM) image of SGA.

The fabrication procedure of the cylindrical SGAs is illustrated in (Fig. 1a). Briefly, a condensed sponge is immersed in GO solution and partially reduced by hydrothermal processing. Simultaneous compression and freezing, to combine chemical self-assembly and mechanical pre-loading treatments, was conducted to integrate the microstructure of the GA with the fractured sponge network. Ice crystals grew and expelled the partially reduced GO (PRGO) sheets to form a 3D linked porous network during the freeze-casting process. This PRGO gel with enhanced strength and elasticity is used to create the sponge-reinforced composite, and the improved mechanical properties allow the cellular GA to survive freeze-drying more easily. The freezing temperature and number of oxygen-containing groups in the GO were found to affect the morphology of this reduced graphene porous structure^8^. After freeze-drying, the ice crystals sublimated, and the rGO sheets as basic building blocks assembled into a fractured PUS network. A schematic (Fig. 1b) showing typical microstructural variations, in which periodic hexagons represent porous sponge array. Through radial compression, the 3D sponge microfibers were deformed into a dense and fractured network. After the sponge-reinforced composite was exposed to a combined freezing and additional reduction treatment, the rGO sheets formed a micro-porous network among the void spaces of the PUS cell array, and all the networks along the macrocellular skeleton formed an hierarchical porous nanocompostie. Figure (1c) shows representative scanning electron microscopy (SEM) images of the SGA. From Fig. (1d), we can see that SGA exhibits a hierarchical skeleton similar to that of pure CGA, indicating that rGO is mainly located within the network instead of coated on the sponge skeleton. The hierarchical structure integrating the porous architecture into the sponge skeleton and the continuous and nearly circular pore structures (5–10 *μ*m diameter) are easily observed. Besides the similar cell dimensions, high-resolution TEM analysis(Fig. 1e and Fig. S5) showing the edge folding of few-layer graphene, the morphology is quite consistent with the previous report^8^.

### Characterization of the SGA

**Figure 2:**
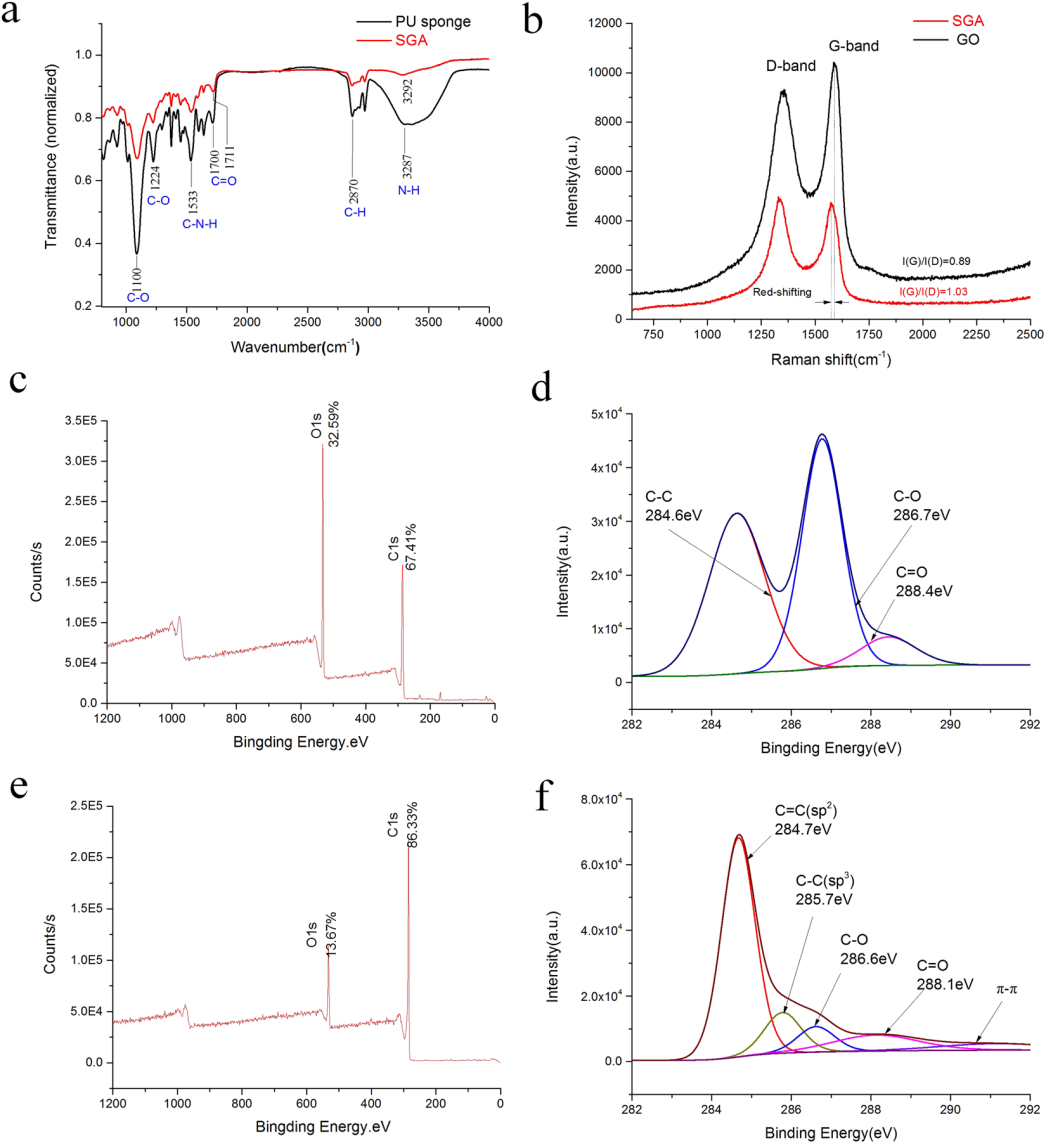
(**a**) FTIR spectra of PUS and SGA; (**b**) Raman spectra; (**c**,**e**) XPS survey scan of GO and SGA; (**d**,**f**) XPS spectra (C1s) of GO and SGA.

The normalized Fourier transform infrared (FT-IR) spectra of PUS and SGA are presented in (Fig. 2a). For PUS, the peaks at approximately 3280, 1700, 1533 and 1100 cm^−1^ are assigned to the stretching vibrations of N-H, C=O, C-N-H and C-O, respectively, which agree well with other PU spectra in the literature^24^. Additionally, most of the characteristic peaks in both spectra are between 800 and 2000 cm^−1^. These similar peaks are expected because SGA and PUS have some similar chemical groups derived from the urethane linkage in their polymer structures, which include carbonyl, amine and amide groups. Nevertheless,the normalized FTIR spectrum of SGA lacks strong peaks compared with that of PUS, which indicates a decrease in the number of oxygen-containing functional groups and the successful reduction of GO to rGO.

Raman spectroscopy was utilized to investigate the degree of disorder of the sp^2^ carbon domains in our graphene material. Figure (2b) shows the Raman spectra of the GO and SGA. The two characteristic peaks can be ascribed to the D-band and G-band, which are associated with the breathing modes in carbon rings and the in-plane vibrations of sp^2^ atoms, respectively^27^. The intensity of the scattering band further elucidates the defective graphene-like structures in proportion to the number I(D) and area I(G) of defects^28^; thus, the I(D)/I(G) intensity ratio can characterize the defect density in the sp2 network. The ratio increased from 0.89 for GO to 1.03 for SGA, presenting basically the same trend as the typical PU/RGO nanocomposite^29^. Compared to the lower I(G) for SGA indicates that additional, smaller sp^2^-bonded carbon structures were formed after the hydrothermal reduction. The small red-shifting of the G band from 1589 cm^−1^ to 1580 cm^−1^ confirms that the GO on the spongy framework was successfully reduced to rGO.

The variation in functional groups and elemental composition were studied by X-ray photoelectron spectroscopy (XPS) peak-splitting analysis to further confirm the reduction of GO sheets during the self-assembly process. In the C1s scan spectra (Fig. 2c and e), the two peaks at 286 and 533 eV correspond to C and O elements. The C/O ratio increased from 2.1 for GO to 6.3 for SGA, due to increased number of C elements and reduced number of O elements. In fact, the elemental O content (13.69%) was close to those chemically reduced GO^7,8^, indicating the deoxygenation of most of the oxygen-containing groups. In (Fig. 2d), the GO spectrum was deconvoluted into three peaks with different binding energies of 284.6, 286.7 and 288.4 eV, corresponding to C=C/C-C in the aromatic rings, C-O in the alkoxyl and epoxyl groups, C=O in the carbonyl groups, respectively. The relative atomic percentages of the C-O and C=O groups in the rGO sheets (6.88 and 9.85%) were significantly lower than those in GO (31.54 and 5.33%). The new peak centered at 291.1 eV is related to the *π*-*π* stacking of C=C/C-C in graphite. These results indicate a high degree of reduction in SGA.

### Mechanical properties of SGA

**Figure 3:**
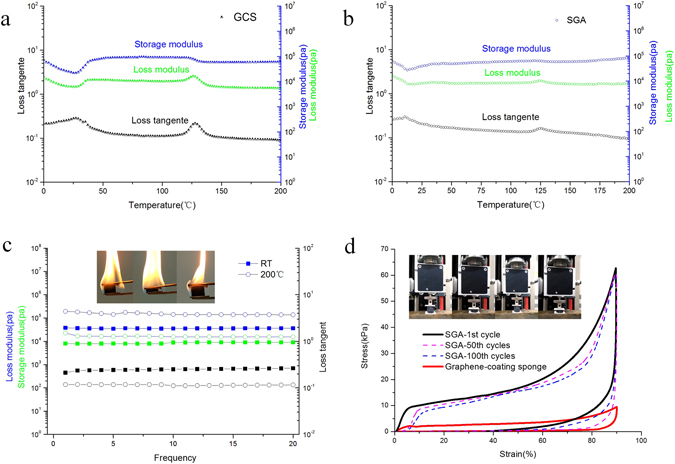
Storage modulus (E’), loss modulus (E”), and tan *δ* (the ratio of E’/E”) as a function of temperature for (**a**) GCS and (**b**) SGA. (**c**) Storage modulus (blue), loss modulus (black) and damping ratio (green) of the SGA is shown as a function of compression frequency at 0–200 °C; The inset images show a burning SGA sample (up to 350 °C) with its typical resilience. (**d**) The compressibility of SGA.

Although the effects of soft and hard segments on the ductility and toughness of the bulk material have been considered in the modeling of cellular PU/CNT composites^30^, the viscoelastic behaviors of these carbon-reinforced nanocomposites have not been adequately investigated^31^. Figure (3a) shows the DMTA results for a typical viscoelastic state (0–200 °C); two sharp peaks of the loss tangent occurred at approximately 27 °C and 126 °C, corresponding to the glass transition temperature (T_g_) of GCS. In the cross-linked networks of the segmented GCS, the T_g_ is determined by the degree of phase separation, i.e. the arrangement of soft and hard segments^32^. Notably, the intensities of the peaks in SGA at the Tg are reduced, exhibiting long plateaus (20–200 °C) with increasing temperature and showing slight maxima than those of GCS during the glass transition, as shown in (Fig. 3b). Therefore, the storage modulus and loss modulus of the SGA are not as dependent on temperature as those of GCS, indicating a broadening of the glass-transition process. SGA, with its increased porosity and networked architecture, becomes more rubbery. This fact and the decreased height of the tan *δ* peak may indicate the formation of more thoroughly cross-linked networks with decreased energy absorption capacity, compared to sponges that do not contain GAs. For examination under 50% dynamic strain at a frequency of 20 Hz, the sample was equilibrated at 25 °C for 2 min and then heated to 200 °C at a rate of 5 °C/min. At the end of the dynamical mechanical test process, SGA was subjected to 42,000 deformation cycles. Notably, the storage and loss moduli values remain nearly constant (Fig. 3c) consisted with the reported G-elastomer^31^. In contrast to the entropic elasticity of ordinary elastomers^33,34^, frequency-dependent viscoelastic properties were observed even at higher temperatures of 200 °C, as show in (Fig. 3c). Such viscoelastic temperature- and frequency-invariant stability, reflects the elastic nature of SGA, making this material appropriate for reusable oil absorption. A combustion flame test (in petroleum ether and air) revealed satisfactory fire resistance, as shown in Supplementary Movie 1. The high compressibility of the SGA was maintained even after burning for at least 25 s. Thus, the SGAs are promising oil sorbents with good recyclability upon burning or compression.

Axial compression testing of SGA with a density of 24 mg cm^−3^ revealed its good rebound resilience (Fig. 3d). In the first cycle (black curve), the stress abruptly increased, demonstrating elastic strain changes of less than 10%. The elastic bending and shearing deformation of the graphene sheets provide the initial compressive strength. The stress increased linearly because of the increasing compaction of the graphene cell walls^35^. As cycling continues, the stress path is repeated (indicated by dotted lines), and three deformation regions occur in the stress–strain curve: (1) a nearly linear elastic region (*ɛ* ≤ 10%)corresponding to the bending of the cell walls, (2) a relatively flat stress plateau(10% ≤ *ɛ* ≤ 70%) corresponding to the elastic buckling of the PUS bonds and the dense and fractured sponge network; and (3) an abrupt increase in stress (70% ≤ *ɛ* ≤ 90%) corresponding to the vertical stacking of the cells, and the SGA sample reached a maximum compressive strength of 63 kpa. In contrast, non-linear elasticity occurs in GCS as a result of the chain segment and phase structure of the PUS. The bottom stress–strain curve shows that the maximum stress of GSC is much lower than that of SGA, suggesting that the porous structure in the aerogel reinforces the elastic stress and yields high compressive deformation, which affects the mechanical properties.

### Absorption properties of SGA

**Figure 4:**
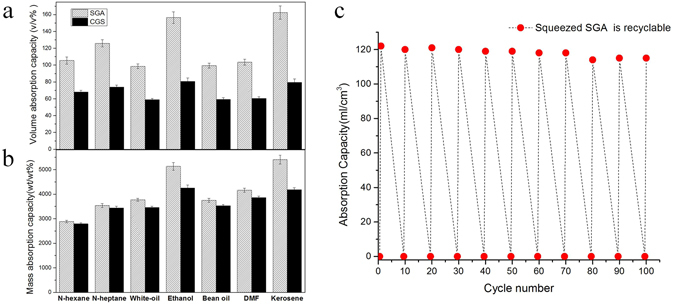
Comparison of the absorption capacities of different organic liquids (kerosene, DMF, bean oil, Ethanol, White-oil, N-heptane and N-hexane) on a (**a**) mass basis and (**b**) volume basis (**b**), error bars show the standard deviations on triplicate measurements. (**c**) The high absorption recyclability of SGA with N-heptane; The inset image shows the spontaneous absorption performance of SGA, in which N-heptane was stained with Sudan Red 3 and floated on deionized water.

The oil-absorbing experiments demonstrate that SGA can absorb a variety of organic solvent with different surface tensions and densities, including kerosene, DMF, bean oil, ethanol, white-oil, n-heptane and n-hexane. A good oil absorption ability, absorbing oil up to 29–54 times its own mass, are higher than that of previously reported porous materials such as nanocellulose aerogels (20–40 times)^36^, PU sponges (15–20 times)^37^, organic nanocomposites^38^, but are close to spongy graphene monolith (20–86 times)^39^, graphene/CNT foam (80–100 times)^40^. Moreover, it shows better oil-absorption capacity than GCS in terms of both volume (Fig. 4a) and mass (Fig. 4b). The mass-based oil absorption capacity Q_*m*_ (weight gain) was calculated from the ratio of the mass of the absorbed oil M_*0*_ to the mass of the absorbing material M_*m*_ (Q_*m*_ = M_*0*_/M_*m*_). The volume-based oil-collection capacity Q_*v*_ (volume gain) is given by the equation Q_*v*_ = V_*0*_/V_*m*_ = (M_*o*_*ρ*_*m*_)/(M_*m*_*ρ*_*0*_), where V_*0*_ and V_*m*_ are the volumes of the absorbed oil and absorbing material, respectively, and *ρ*_*m*_ and *ρ*_*0*_ are the densities of absorbing material and oil, respectively. The density of SGA was calculated to be 24 ± 2 mg cm^−3^, which is comparable to or slightly lower than that of some aerogel composites^41^. Since Q_*m*_ is strongly affected by the density of absorbing materials and oils^42^, the volume-based absorption capacity (Q_*v*_) was also used to assess the performance of the prepared low-density material. Compared to previously reported ultralight graphene-based sorbent, SGA shows efficient space utilizations for oil absorption. Although the Q_*m*_ (weight gain) is lower than that of ultralight counterparts, the volume ratio of absorbed liquid is much higher, as shown in Table 1. The absorption capacity of SGA exceeds 1 mL cm^−3^, which is attributed to the combination of high porosity and surface hydrophobicity, as well as the polymer swelling effects^44^. The capillaries of the superoleophilic microtubes are perceived to drive the oils into the pores of the sponges^42,45^. Therefore, oils can permeate into the void space in the PU skeleton and be stored in the inner micro pores of SGA, contributing to a much higher Q_*v*_ than that of GCS and other superhydrophobic foams.

**Table 1:** Oil absorption capacity (weight gain and volume gain) of various sorbent materials.

Materials	Density	Weight gain(g g^−1^)	Volume gain%(v/v)
Carbon ultralight weight aerogel^43^	0.75 mg cm^−3^	350	25.6% for crude oil
Hybrid graphene/CNT foam^40^	6.92 mg cm^−3^	80–100	68.3% for compressor oil
Ultralight Fe_2_O_2_/C foam^42^	8.9 mg cm^−3^	60–100	70% for decane
Spongy graphene monolith^39^	12 mg cm^−3^	20–86	40% for heptane
Graphene/PU Sponge^24^	8.8 mg cm^−3^	70–100	68.5% for diesel oil
Functionalized graphene aerogel^6^	14.4 mg cm^−3^	50–112	73% for acetone
Spongy graphene aerogel (this study)	24 mg cm^−3^	29–54	125% for N-heptane

**Figure 5:**
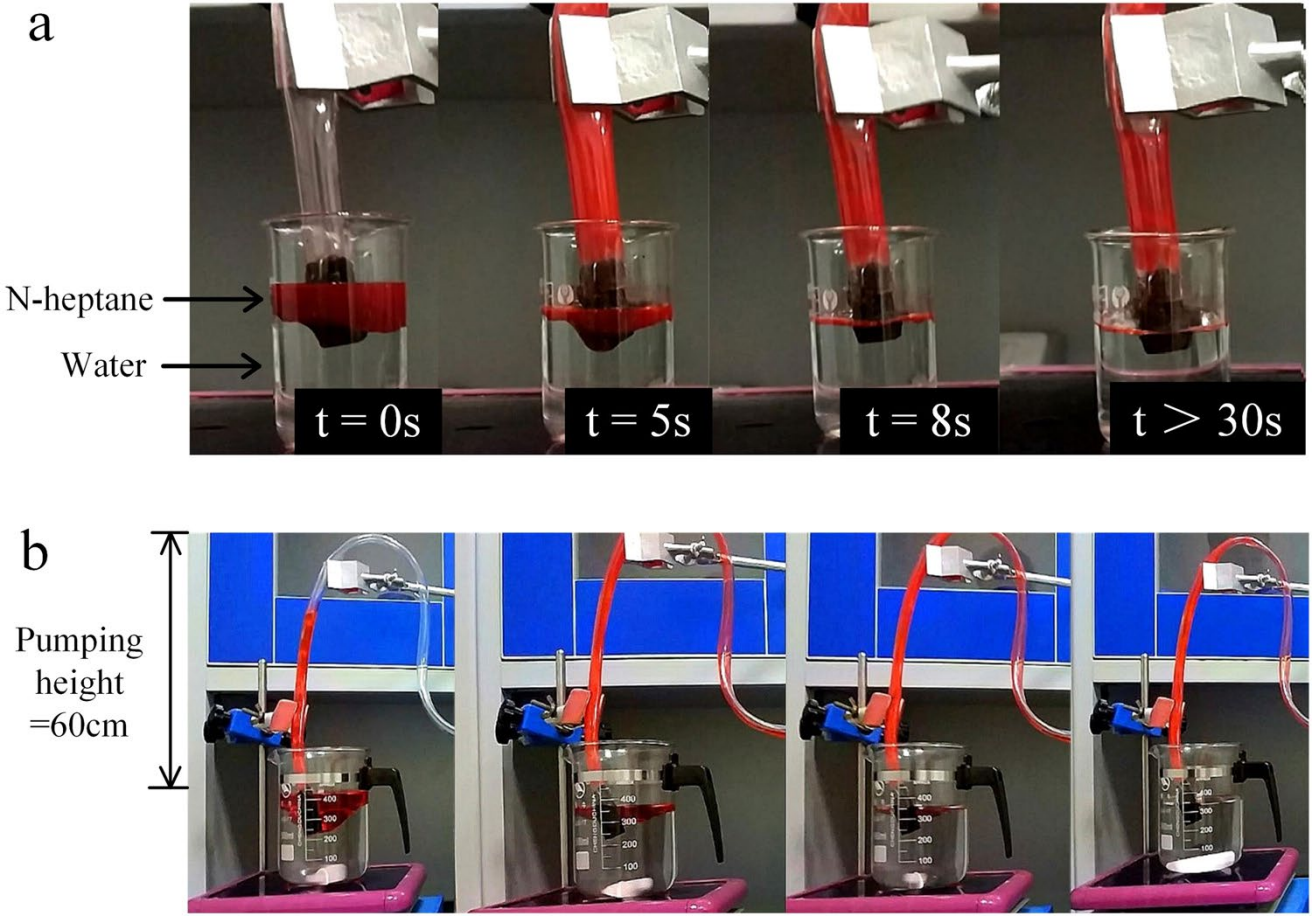
(**a**) The selective absorption of SGA in an oil-water mixture with pumping action. (**b**) The continuous removal of n-heptane using a 120-W pump. N-heptane was stained with Sudan Red, and the pumping height was 0.6 m (as shown in Supplementary Movie 3).

The high resilience and compressibility of SGA were maintained over numerous cyclic absorption/evacuation cycles, indicating that this oil-removal process is much more efficient than burning and distilling for oil removal. Thus, we evaluated the reusable performance of our material over 100 absorption/evacuation cycles with n-heptane, and the results are shown in (Fig. 4c). The highly repeatable absorption/evacuation process indicates the sustained and high absorption performance of our sponge material, as well as the structural stability and recoverability of the material for mechanical oil remediation. The batch absorption process of n-heptane removal is shown in (Supplementary Movie 2). Although highly saturated oil samples were manually removed, the inefficient compression evacuation approach is impractical for continuous use. To treat large-area oil spills, the 3D porous structure provides space to store and then evacuate the absorbed oil in a continuous absorption process. Thus, the high-efficiency removal of oil from water can be achieved using an oil pump. For these experiments, one end of a cylindrical SGA sample was submerged into the oil-covered water while the inlet pipe of an oil pump was placed at the opposite end. When the pressure-driven system is operated, capillarity and selective absorbability work in tandem to load the high-volume absorber with oil, which is rapidly extracted upwards through the pipe, as shown in Fig. 5a). The absorption process generally reached equilibrium within 8 s (as shown in Supplementary Movie 3), which is faster than previously reported removal times of 30 s^3^. The stable water level in the beaker with prolonged continuous pumping indicated that water was not removed by SGA.

Compared to stationary water in a beaker, real environmental conditions usually involve oceanic disturbances during practical applications associated with oil-contaminated water. Therefore, as an example of a practical application, another experiment was performed in which a magnetic stir bar was used to stir the water to imitate ocean conditions. In a wide-mouth beaker, 100 mL of n-heptane was stirred into a rotational vortex, with air bubbles flowing upward. Notably, the pumping height was 0.6 m, which better reflects the vertical height difference between the sea surface and a ship than the end-to-end distance of the experiment. The n-heptane vortex was continuously removed from the water using a low-power oil pump, as shown in Fig. 5b. The water in the beaker was unaffected, and the entire process lasted for only a few minutes (as shown in Supplementary Movie 4). Moreover, n-heptane continuously added to the beaker was removed by pumping. The general continuous absorption rate was 1800 liters per square meter per hour (LMH), and the separation efficiency was over 99.9% after 24 h of separation. These results demonstrate the recoverability and selective oil-absorption capacity of SGA for continuous absorption in practical applications.

**Figure 6:**
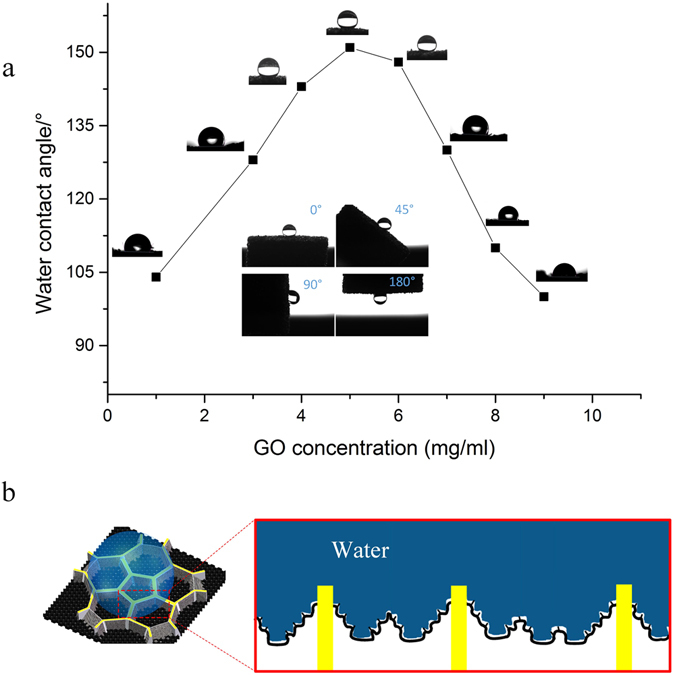
Wettability of SGA. (**a**) Variation of the WCAs with the concentration of GO solution; Inset image shows the water droplet on a rotated SGA. (**b**) Schematic diagram of a water drop in contact with the composite.

In the WCA studies, both pure GAs and PUS showed better water wettability than SGA. Because of the surface roughness and the presence of heteroatoms distributed on the surface, the WCA of pure GAs was approximately 100° ^9^. PUS synthesized by direct polymerization also possessed a certain hydrophobicity and exhibited a similar WCA, as the long PUS chains can effectively suppress surface reorganization upon contact with water. Integration of the macroscale PU framework and microscale graphene scaffold led to enhanced hydrophobicity, as reflected by the increased WCA (as shown in Fig. 6a). When the GO precursor concentration was approximately 5 mg/ml, the WCA was significantly increased, and a superhydrophobic surface (WCA of 152°) is achieved. With a further increase in the GO concentration, the WCA decreased due to the poorly organized structure of the resulting PUS/GA, in which the blocking GA pores were unevenly distributed throughout the sponge skeleton. We thus conclude that the hierarchically rough surface of SGA produced the resulting surface superhydrophobicity. The inset in Fig. 6a shows that the surface of SGA exhibited water-adhesive hydrophobicity during the rotation test, in which a small 20 μL water droplet adhered to the sample surface. Based on the internal porous structure and the presence of deep wrinkles on the graphene walls, the droplet can be expected to partially permeate into the pores of PUS/GAs without immediate contact, leading to the unusual Cassie impregnating wetting state^46^ (as illustrated in Fig. 6b). This state can produce a “rose petal” effect in which the partially sealed droplet is tightly pinned to the surface due to the high adhesion energy, which was responsible for the high CA hysteresis of SGA^47^. Thus, the wetting behavior of SGA, i.e., its large contact angle, adhesion, and high CA hysteresis, can further guide the design of biomimetic superhydrophobic surfaces.

## Conclusion

In summary, spongy GAs with superhydrophobic durability and facile recyclability were successfully prepared through a novel and promising synthetic strategy. The enhanced strength against axial compression and stable viscoelasticity over repeated compression cycles show that the resultant porous sorbent is thermally and mechanically stable and potentially capable of the *in situ* remediation of oil spills in harsh environments. Optimizing the rGO self-assembly process with a pre-compaction step and controlling the internal nano/microstructure with hierarchical pores simultaneously led to the high oil-absorption capacity and excellent recyclability. Furthermore, SGA accomplished the continuous selective separation of oil/water mixtures under pumping action. Consequently, the demonstrated SGA, with its unique wettability, is not merely an efficient reusable absorbent for use in traditional desorption methods, but it can also be used in the continuous removal and absorption of oil or organic solution from the surface of water. We believe that the monolithic structure developed using this clean synthetic strategy holds considerable promise for new applications in sustainable petroleum contamination remediation.

## Methods

### Preparation of GO

GO was prepared by the oxidation of purified natural graphite (Sinopharm Chemical Reagent Co., Ltd.) based on the improved Hummer’s method^48^. A 500-mesh graphite flake (3 g) was dissolved in 100 mL concentrated sulfuric acid (95%) in an ice bath under magnetic stirring (100 rpm). Then, potassium permanganate (13.5 g) was added slowly to prevent the temperature from going over 20 °C. The reaction continued for 1 h at 40 °C with vigorously stirring (800 rpm). Then, 750 mL of water was added slowly and the solution was stirred at 90 °C for 30 min. After the addition of 500 mL of deionized water, the cooled acid mixture was further treated with a hydrogen peroxide solution (800 mL, 5%). The mixture was washed with distilled water and 300 mL of hydrochloric acid (10%) followed by repeated washing with ethanol. The resultant bright yellow suspension was purified by repeated centrifugation and washed with distilled water and ethanol until reaching pH 7.0. Finally, the filtrate liquid was air dried overnight and sonicated for 30 min to exfoliate it to create the GO dispersion.

### Preparation of GCS

In a typical procedure, recycled PUS samples (obtained from Wuhan Litai Chemical Co., Ltd) with cubic (50 mm × 15 mm × 15 mm) and cylindrical (10 mm diameter and 50 mm height) morphologies was washed with ethanol in an ultrasonic cleaner. To prepare the GCS, the cylindrical PUS samples were dip-coated in 5 mL of the as-prepared GO suspension by manual compression. The PUS/GO compound was mixed with ascorbic acid (1:10, w/w) in a test tube, which was then directly heated at 95 °C for 8 h. Gelation of the compound occurred within 2 h, and a nearly homogenous gel-like material was obtained. After 8 h of hydrothermal reduction to achieve the complete removal of oxygen content, the gel was freeze-dried in a Christ ALPHA 1–4 freeze-dryer (Germany). Thus, the final GCS with reduced GO (rGO) sheets was obtained from the assembly of graphene nanosheets on PUS.

### Preparation of SGA

The GO solution (5 mg/mL) was mixed with ascorbic acid (1:10, w/w) under ultrasonic agitation for 15 min at temperatures below 25 °C before use. The cubic PUS was loaded in a 10-mm-diameter Teflon mold with the aid of tweezers (33% strain in the radial direction). The mold was submerged in a test tube containing the GO suspension. The residual gas in the sponge was removed by exposure to vacuum for 30 min. After heating the test tube in a 95 °C oil bath for 15 min, the PUS/GO mixture was partially reduced to an intermediate, slightly shrunken, homogenous material. Subsequently, simultaneous compression and freezing was performed by cooling the mixture in the mold in a dry ice/ethanol bath. The nested pore structure was realized by the freeze-casting of the gel-like sponge in the compressed state. After subjecting the compressed sponge to reduction for another 8 h at 95 °C, the softened sponge composite with a new configuration was obtained. Finally, the sponge was freeze-dried to sublimate the ice crystals contained in the sponge skeleton to prepare the robust and hydrophobic SGA with a porous microstructure.

### Characterization

GO and SGA were characterized using a Thermo VG RSCAKAB 250X high-resolution X-ray photoelectron spectroscope (XPS) and a Nicolet 6700 Fourier transform infrared (FTIR) spectroscope. The microstructure and morphology were observed using a Hitachi SU8010 scanning electron microscope (SEM) at 20 kV. TEM observation were performed with a FEI, Tecnai G2 F20 TEM at 250 kV. Raman spectra were measured using a Renishaw spectrometer (RM-1000) with a 532 nm laser (20 mW). DMTA was conducted using a dynamic mechanical thermal analyzer (Diamond DMTA, PerkinElmer) with test specimens 6 mm in length and 10 mm in diameter, cut from the prepared SGA. The storage and loss moduli and loss tangent as a function of frequency were measured with 50% pre-strain and oscillatory compressive strain. The stress–strain curve of SGA was determined using a Zwick table-top testing machine (Z010 TH) in strain control mode with a compressive strain rate of 90% min^−1^. The WCAs of the samples were measured using a DSA30 drop shape analyzer (KRUSS Instruments).

## Electronic supplementary material

Supplementary information

Supplementary movie 1

Supplementary movie 2

Supplementary movie 3

Supplementary movie 4
