# Publisher Correction: Highly reusable and superhydrophobic spongy graphene aerogels for efficient oil/water separation

**DOI:** 10.1038/s41598-017-18752-6

**Published:** 2018-01-05

**Authors:** Yuanzheng Luo, Shenlin Jiang, Qi Xiao, Chuanliang Chen, Buyin Li

**Affiliations:** Key Laboratory of Electronic information functional material of Ministry of Education, School of Optical and Electronic Information, Huazhong University of Science and Technology, Wuhan, Hubei 430074 China

Correction to: *Scientific Reports* 10.1038/s41598-017-07583-0, published online 02 August 2017

The PDF version of this Article was inadvertently replaced after publication. This has now been corrected; the HTML version of the paper was correct from the time of publication.

