# Supplementary material for: Highly reusable and superhydrophobic spongy graphene aerogels for efficient oil/water separation
Source: Sci Rep. 2017 Aug 2;7:7162. doi: 10.1038/s41598-017-07583-0 (PMC5540914; doi:10.1038/s41598-017-07583-0)
Supplement: Supplementary file 1 — Supplementary information [file 41598_2017_7583_MOESM1_ESM.doc]

**Highly reusable and superhydrophobic spongy graphene aerogel for efficient oil/water separation**

Yuanzheng Luo, Shenglin Jiang, Qi Xiao, Chuangliang Chen, Buyin Li*,

Key Laboratory of Electronic information functional material of Ministry of Education, School of Optical and Electronic Information, Huazhong University of Science & Technology, Wuhan, Hubei 430074, China

**Table of contents**

1. Fig. S1: Digital macro photos and a section of SGA.

2. Fig. S2: X-Ray Diffraction of GO and SGA.

3. Fig. S3: The morphologies of the SGA and compressible graphene aerogel(CGA)

4. Fig. S4: The morphologies of the SGA and graphene coating sponge(GCS)

5. Fig. S5: Transmission electron microscopy(TEM) images of SGA.

6. Fig. S6: Thermogravimetric (TG) and differential thermal analyses (DTA) of the SGA

7. Fig. S7: Contact angle variation during 200 recycle numbers

8. Fig.S8: A comparison of the microstructure of SGA and GCS after burning

8. Supplement movie 1-4

9. References

Corresponding author*. Tel.: 13476124828; 027-87542994.

E-mail address: 59004796@qq.com; yuanzluo@163.com

1. **Digital macro photos of SGA**

**
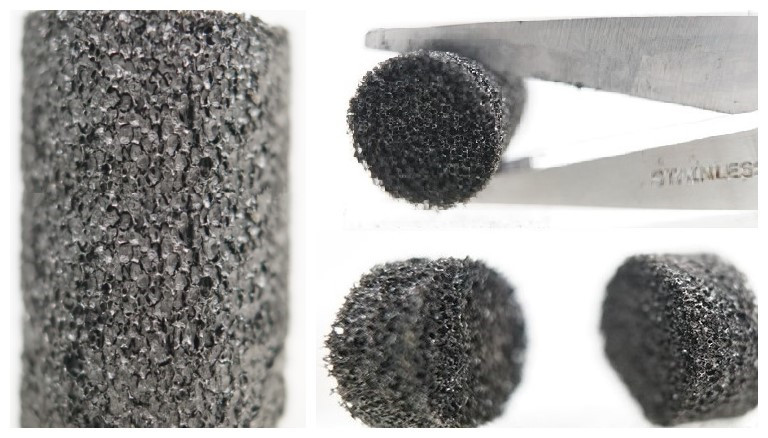
**

**Fig.S1** Macro photos of cylindrical SGA. To confirm that the rGO sheets could interpenetrate the sponge, sample was cut into pieces as shown.

**2. X-Ray Diffraction of GO and SGA.**


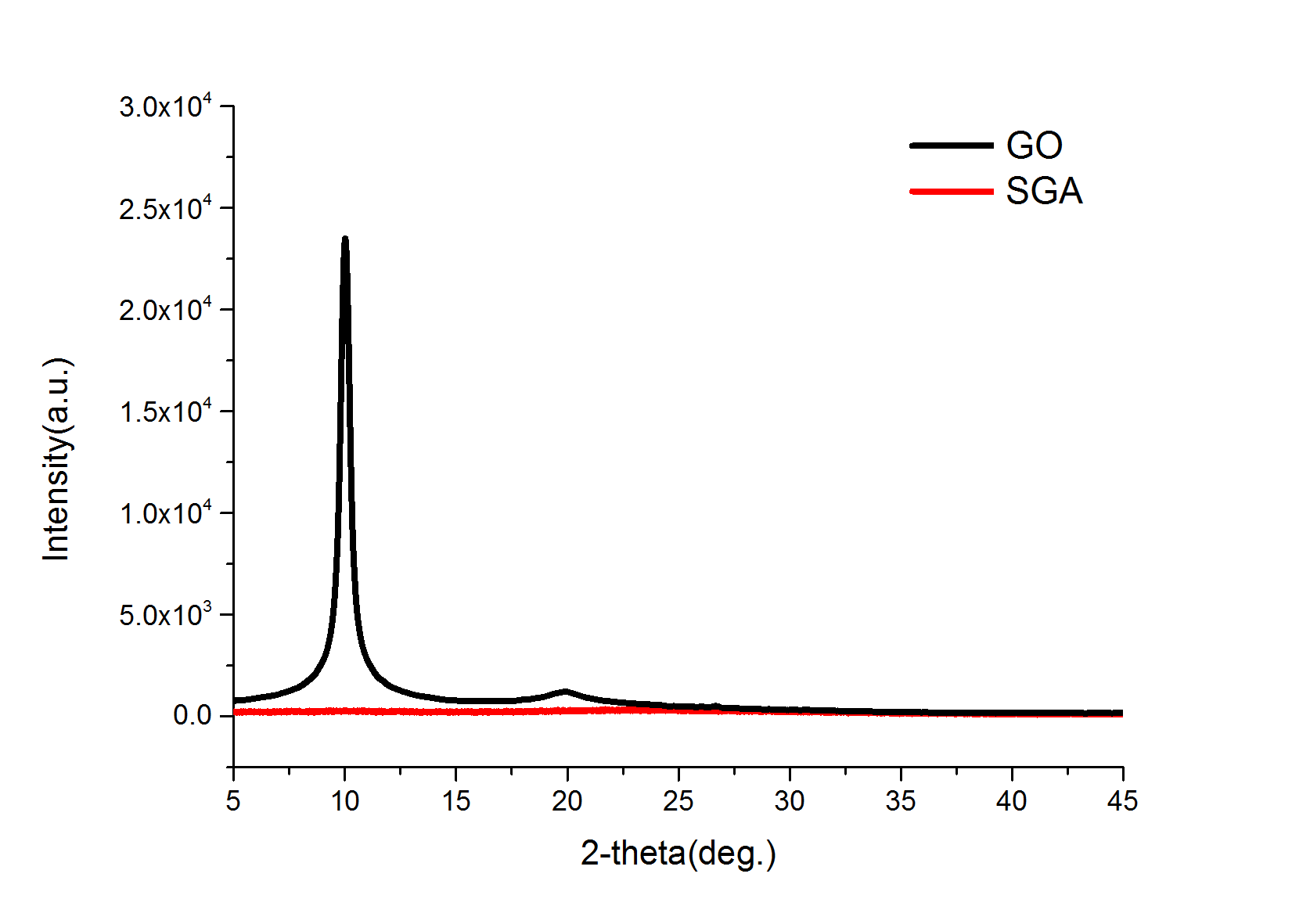


**Fig.S2** The XRD patterns of GO and SGA.

The phases structural of the obtained GO and SGA were studied by XRD, the results are shown in Fig.2a). The diffraction peaks at 2θ=10.8° disappears in the XRD pattern of SGA, indicating there is no three-dimensional stack of the reduced GO (RGO) sheets.

**3.** **The morphologies of the SGA and compressible graphene aerogel(CGA)**

**
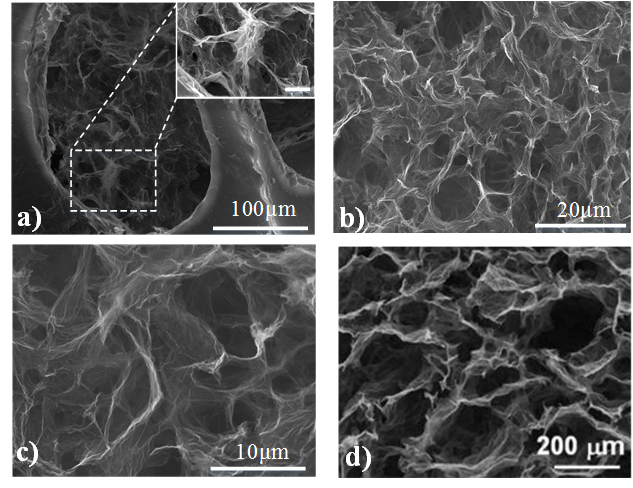
**

**Fig.S3** The similar hierarchical structure found in SGA is similar to the previously reported CGA^[1]^. a),b) and c) are SEM images of SGA in different resolution. d) SEM image of CGA.

**4.The morphologies of the SGA and graphene coating sponge(GCS)**

**
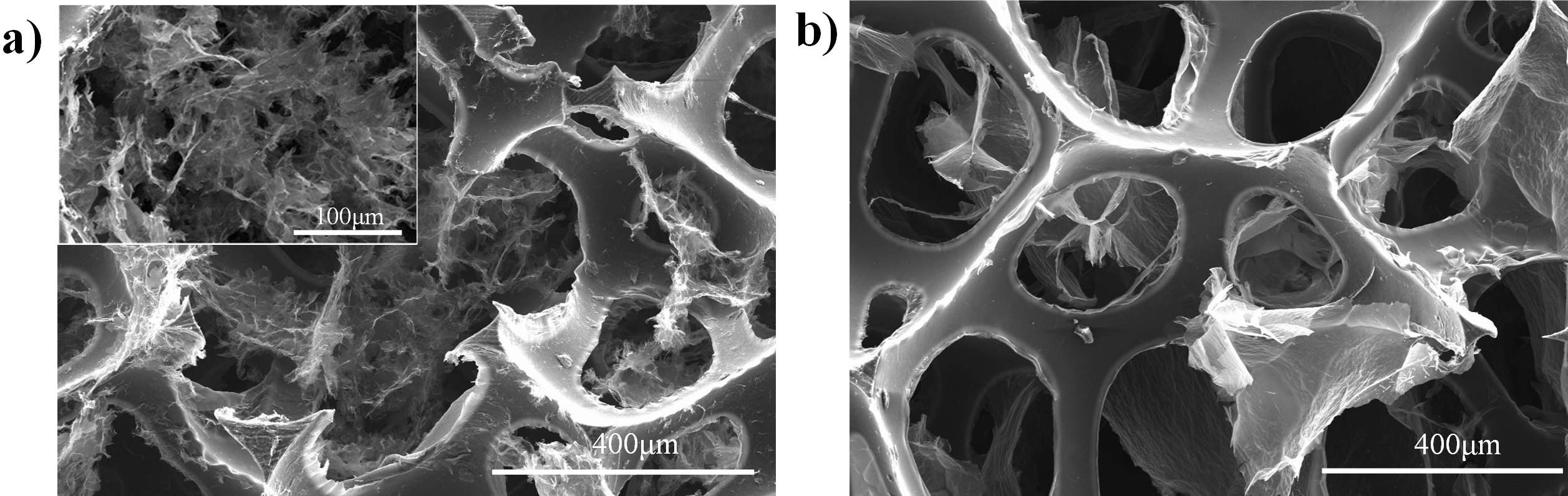
**

**Fig.S4** a) SEM image of the porous structure of SGA; Inset image is the high-magnification image of SGA showing the structure of the porous b) SEM image of the graphene-coated sponge

The hierarchical porous structure of SGA is demonstrated from a microscopic perspective by SEM in Fig.S3 a). The nearly spherical pores are evenly distributed into the dense sponge skeleton without obvious aggregation, showing good dispersion of graphene aerogel. By contrasting the microstructure of GCS with that obtained using a dip-coating solution followed by same reduction and freeze drying, as shown in Fig.S3 b), the SEM image shows that transparent graphene sheets cover half the sponge network. Therefore, unlike the coating process of a conformal graphene “skin” on the surface of the PU skeletons, the combination of pre-loading design and a low-cost wet-shaping technique presents a simpler and greener route to form a stable nested porous structure, which corresponded to the oil absorption behavior.

**5. Transmission electron microscopy(TEM) images of SGA.**


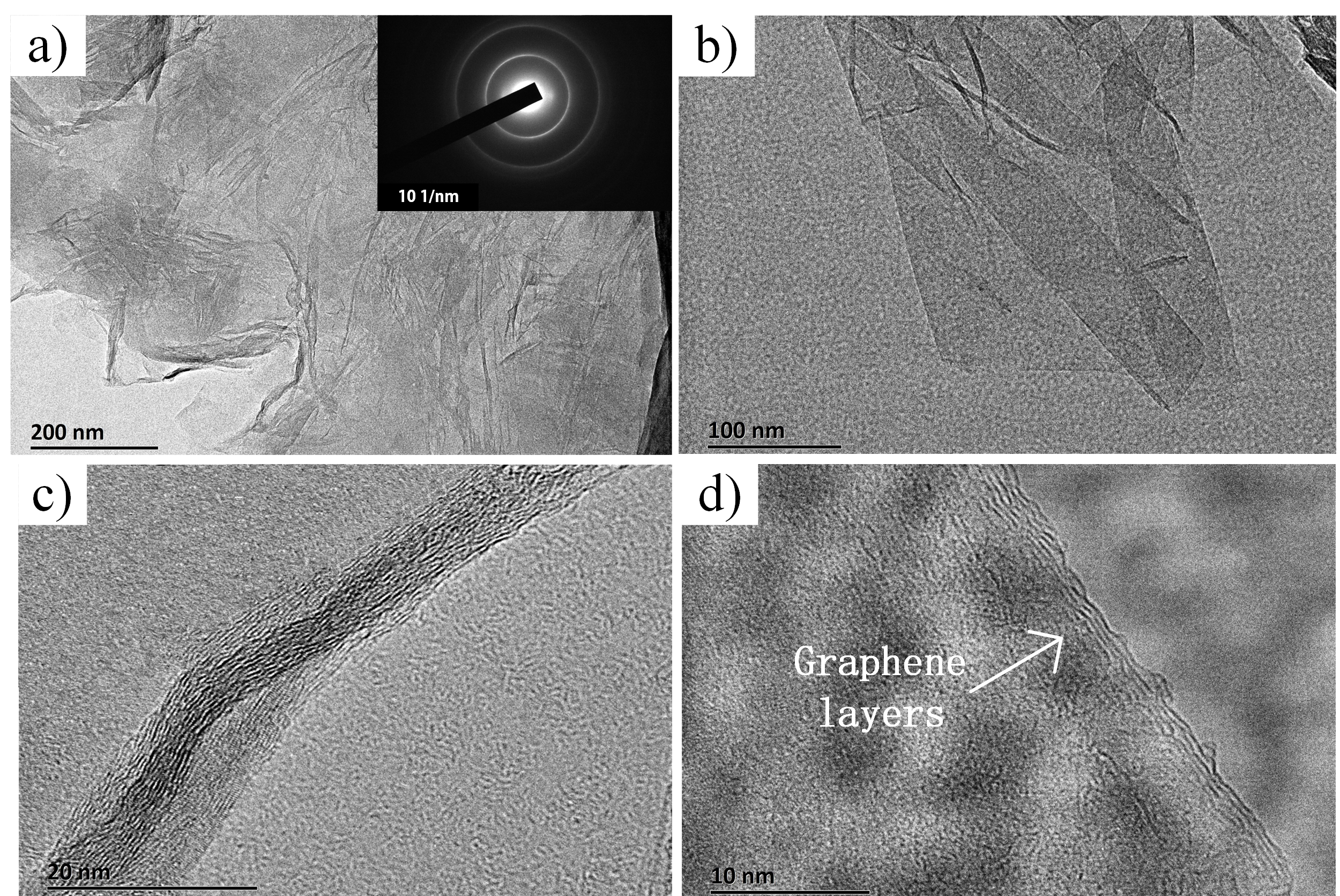


**Fig. S5** The high-resolution TEM images of SGA. (a) TEM image of SGA on a microgrid. The insert is the selected area electron diffraction pattern. (b)TEM image of the apparent graphene sheets. (c) TEM images showing the edge folding of few-layer graphene, the morphology is quite consistent with the previous report^[2]^. (d) The HRTEM shows well-defined lattice fringes of stacked graphene (white arrow).

**6. Thermogravimetric (TG) and differential thermal analyses (DTA) of the SGA**

**Fig.S6** TG-DTA curve of SGA in nitrogen atmosphere.

In the obtained TG results, the loss curves of weight SGA degrade following a two-step process, due to the decomposition of hard and soft segments in polyurethanes sponge. The DTA curve was decreases steadily without decalescence peaks, which means the decomposition rate of the obtained composites is evenness. This thermal stability of SGA is very different from organic matter and graphene/thermoplastic polyurethane foam^[3]^ due to the unique graphene structure. Which indicate the addition of graphene aerogel reinforced thermal characteristics of each component as a whole.

**7. Contact angle variation during 200 recycle numbers**

**Fig.S7.** Contact angle during 200 times separation of oil/water mixture (immerse/squeeze cycles).

**8. A comparison of the microstructure of SGA and GCS after burning**

**
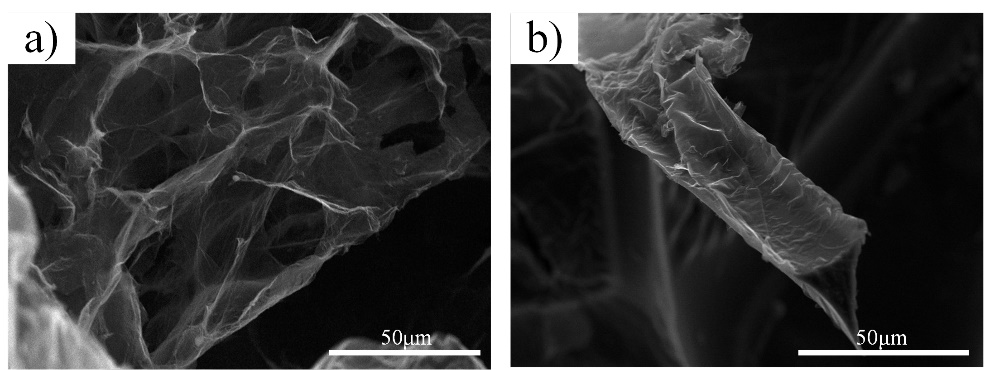
**

**Fig S8.** (a) SEM image of graphene wrapped tube after removing the PU backbone of GCS.(b) SEM image of residual porous and void space after removing the PU backbone.

**9. Supplement movie 1-4**

**Supplement movie 1**. A simple combustion experiment is used to evaluate the flame retardancy of SGA, the compressibility maintained even after 25s burning.

**Supplement movie 2**. Oil absorption test in oil/water mixture under static.

**Supplement movie 3.** A comparison experiment of SGA and natural sorbent(cotton) for the separation of the oil/water mixture in pumping application. The oil/water selective absorption for the removal of oil from the water is of great potential for oil spill clean-up.

**Supplement movie 4**. Continuous oil absorption of SGA with a 200w Electric Oil Pump and 0.6m pump height for the removal of 100ml N-heptane under stirring.

**10. References**

1. Hu, H.; Zhao, Z.; Wan, W.; Gogotsi, Y.; Qiu, J., Ultralight and highly compressible graphene aerogels. *Advanced Materials* **2013,** *25* (15), 2219.

2. Qiu, L.; Liu, J. Z.; Chang, S. L.; Wu, Y.; Li, D., Biomimetic superelastic graphene-based cellular monoliths. *Nature communications* **2012,** *3*, 1241.

3. Liu, H.; Dong, M.; Huang, W.; Gao, J.; Dai, K.; Guo, J.; Zheng, G.; Liu, C.; Shen, C.; Guo, Z., Lightweight conductive graphene/thermoplastic polyurethane foams with ultrahigh compressibility for piezoresistive sensing. *Journal of Materials Chemistry C* **2016**.
